# Isolated Laryngeal Amyloidosis Mimicking Laryngeal Cancer

**DOI:** 10.7759/cureus.3106

**Published:** 2018-08-06

**Authors:** Aeman Muneeb, Supriya Gupta

**Affiliations:** 1 Radiology, Aga Khan University, Karachi, PAK; 2 Radiology, Rush University Medical Center, Chicago , USA

**Keywords:** laryngeal amyloidosis

## Abstract

Amyloidosis is the deposition of an extracellular fibrillar protein in the tissues leading to organ dysfunction. Laryngeal amyloidosis is a rare phenomenon. We report a case of isolated laryngeal amyloidosis which was initially suspicious for laryngeal cancer on magnetic resonance imaging (MRI) but histopathology showed the presence of amyloid. Systemic workup was negative. The patient is being managed conservatively.

## Introduction

Extracellular deposition of an abnormal fibrillar protein in the tissues is termed amyloidosis [1]. The incidence of amyloidosis ranges from 5 to 13 per million per year individuals worldwide [2]. Diagnosis requires histological examination of the tissue with Congo red stain [1].

Amyloidosis most commonly affects men around the age of 50 to 70 years [3]. Treatment is based on timely identification and therapies are aimed at reducing the production of new amyloid protein by limiting the supply of amyloid precursor protein [1].

In the head and neck, the larynx is one of the frequent sites of amyloid deposition [3]. It may involve true vocal cords, false vocal cords or may be transglottic [4]. Isolated laryngeal amyloidosis is a rare phenomenon and comprises about 0.2% to 1.2% of all benign laryngeal tumors [3] Even though laryngeal amyloidosis is a localized process, it may be associated with systemic disease, and the recurrent/persistent disease appears to be the norm rather than the exception [4]. The laryngoscopy findings usually demonstrate a yellowish-white mass arising from the larynx [3,5]. Our case report emphasizes the importance of keeping a high suspicion for laryngeal amyloidosis in a patient with atypical presentation of hoarseness especially slow-growing masses, with characteristic findings on laryngoscopy.

## Case presentation

**Figure 1:**
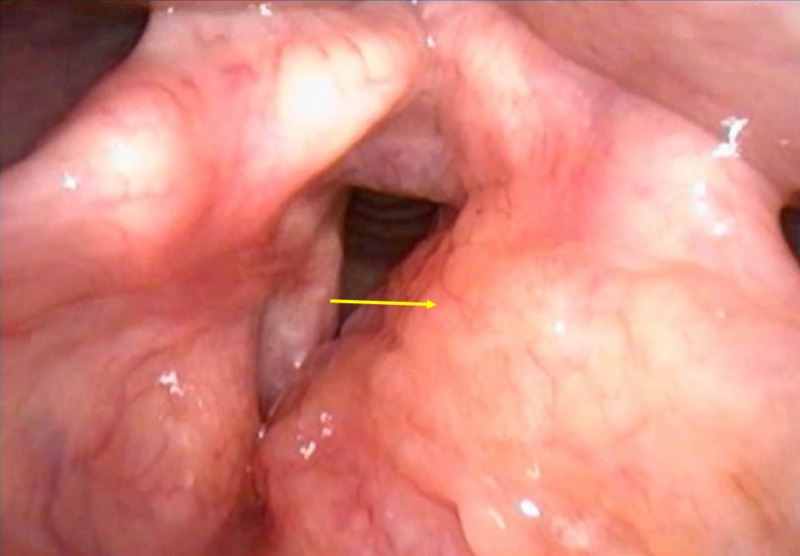
Laryngoscopy findings Left supraglottic/false vocal fold submucosal fullness (arrow) extending to the aryepiglottic fold with papillomatous changes to the mucosa of the false vocal fold.

A 31-year-old male presented with stable dysphonia for four years. There were no sinonasal complaints, dyspnea, dysphagia, sore throat or a cough. Direct laryngoscopy exam showed left supraglottic/false vocal fold submucosal fullness extending to the aryepiglottic fold with papillomatous changes to the mucosa of the false vocal fold (Figure 1). 

**Figure 2:**
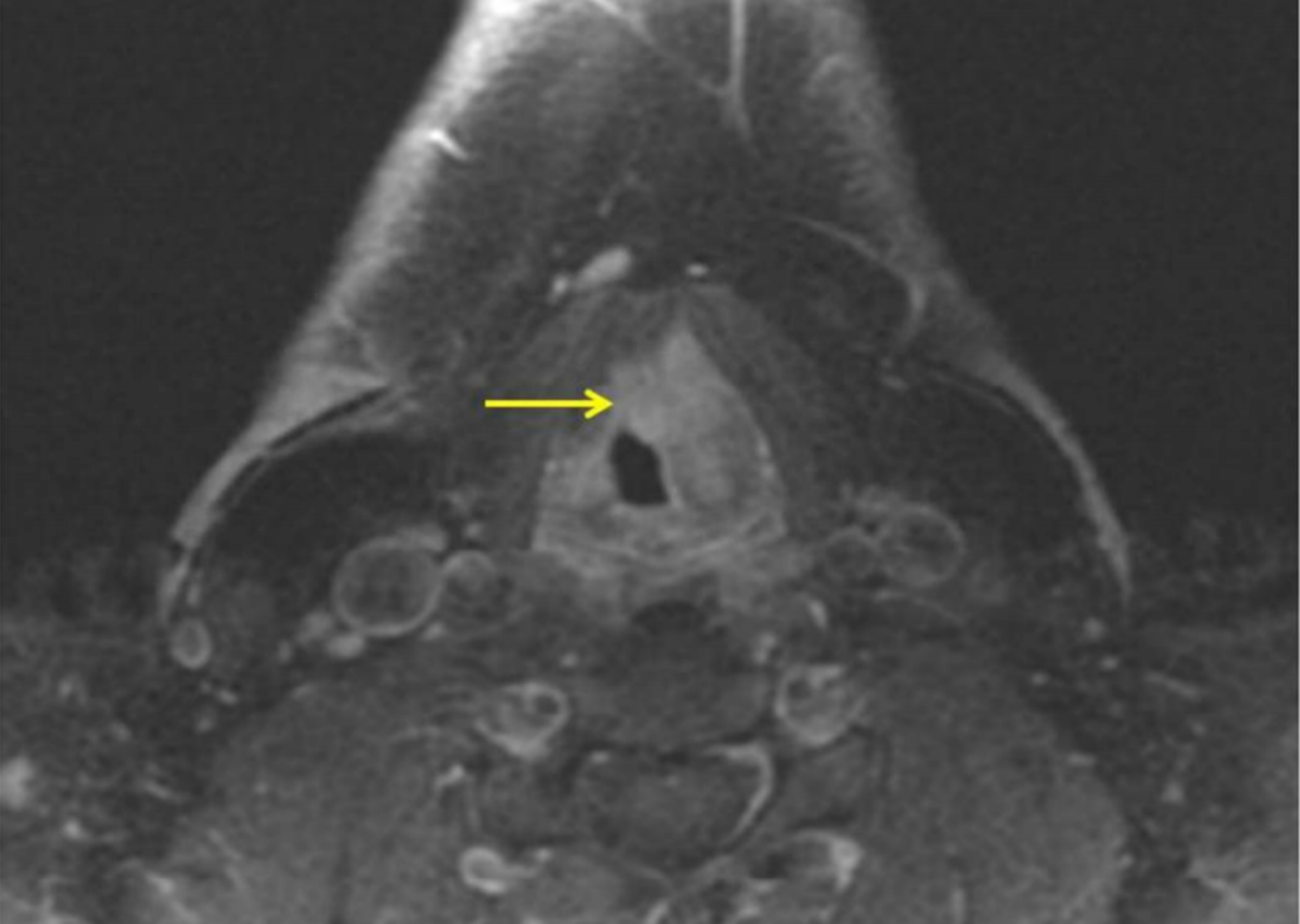
Axial T1 post-contrast magnetic resonance imaging (MRI) Ill-defined enhancing soft tissue mass (arrow) centered in the left false vocal cord and paraglottic fat, extending to the left true vocal cord, anterior commissure, and left aryepiglottic fold.

Magnetic resonance imaging (MRI) of the neck revealed an ill-defined enhancing soft tissue mass centered in the left false vocal cord and paraglottic fat, extending to the left true vocal cord, anterior commissure, and left aryepiglottic fold (Figure 2). Imaging findings were suspicious for laryngeal cancer. No pathologically enlarged lymph nodes were identified by the imaging size criteria. The pathology report showed congophilic amyloid deposits within the stroma and around the sub-mucosal glands containing a mixed population of kappa and lambda staining plasma cells. The findings were consistent with laryngeal amyloidosis. Systemic workup including serum and urine electrophoresis, cardiac exam, and bone marrow biopsy was negative. Local debridement was considered; however, due to the disease size and concern for residual amyloidosis, the patient was managed conservatively. Follow-up MRI performed three months later showed no interval change.

## Discussion

Two possible mechanisms have been hypothesized leading to the development of isolated localized amyloidosis [3]. The first mechanism hypothesizes abnormal proliferation and deposition of plasma cell clones that produce amyloid light chain protein. The second mechanism focuses on the body’s inability to clear light chain proteins resulting in localized deposition.

The most common presentation of laryngeal amyloidosis is hoarseness, even though a cough, hemoptysis, dysphagia or dyspnea may be present [3-4]. A review of 11 cases [4] found that all cases presented with the chief complaint of hoarseness similar to our case. MRI findings were suspicious for laryngeal cancer but pathology report showed amyloidosis. It has been reported that MRI is a generally reliable tool to diagnose amyloidosis since amyloid gives an intermediate T1 signal and low T2 signal [3]. However, since amyloidosis may mimic cancer, the clinician must have a reasonable degree of suspicion and confirm the diagnosis with a biopsy [6].

Laryngeal amyloidosis is most often treated surgically [4]; however, in our case, surgery was avoided due to concerns for incomplete excision and residual disease. Conservative management was undertaken. Recurrent disease is common with a good 10-year overall survival after initial presentation [4]. Some patients may spontaneously go into remission with no evidence of disease years after diagnosis [4].

New therapies such as anti-amyloid antibodies are under trial for systemic disease. These have the ability to dissolve amyloid deposits [7] thus reducing the population of monoclonal plasma cells responsible for amyloid production.

## Conclusions

Amyloidosis of the larynx is a rare disease, often presenting with hoarseness. Clinical suspicion for this entity should be high in young patients with slow-growing laryngeal mass. Treatment currently consists of surgical resection though newer therapies for amyloidosis may lead to better outcomes.
